# Family cascade screening for equitable identification of familial hypercholesterolemia: study protocol for a hybrid effectiveness-implementation type III randomized controlled trial

**DOI:** 10.1186/s13012-024-01355-x

**Published:** 2024-04-09

**Authors:** Christina Johnson, Jinbo Chen, Mary P. McGowan, Eric Tricou, Mary Card, Amy R. Pettit, Tamar Klaiman, Daniel J. Rader, Kevin G. Volpp, Rinad S. Beidas

**Affiliations:** 1 Feinberg School of Medicine, Northwestern University, Chicago, IL USA; 2 Perelman School of Medicine, University of Pennsylvania, Philadelphia, PA USA; 3 Family Heart Foundation, Fernandina Beach, FL USA; 4 Dartmouth Hitchcock Medical Center, Lebanon, NH USA; 5 Independent Consultant, Boston, MA USA; 6 Penn Center for Health Incentives and Behavioral Economics, University of Pennsylvania, Philadelphia, PA USA; 7 The Wharton School, University of Pennsylvania, Philadelphia, PA USA

**Keywords:** Familial hypercholesterolemia, Cascade screening, Health equity, Behavioral economics, Evidence-based practice, Implementation science, Genetic disorders, Hybrid effectiveness-implementation trials

## Abstract

**Background:**

Familial hypercholesterolemia (FH) is a heritable disorder affecting 1.3 million individuals in the USA. Eighty percent of people with FH are undiagnosed, particularly minoritized populations including Black or African American people, Asian or Asian American people, and women across racial groups. Family cascade screening is an evidence-based practice that can increase diagnosis and improve health outcomes but is rarely implemented in routine practice, representing an important care gap. In pilot work, we leveraged best practices from behavioral economics and implementation science—including mixed-methods contextual inquiry with clinicians, patients, and health system constituents—to co-design two patient-facing implementation strategies to address this care gap: (a) an automated health system-mediated strategy and (b) a nonprofit foundation-mediated strategy with contact from a foundation-employed care navigator. This trial will test the comparative effectiveness of these strategies on completion of cascade screening for relatives of individuals with FH, centering equitable reach.

**Methods:**

We will conduct a hybrid effectiveness-implementation type III randomized controlled trial testing the comparative effectiveness of two strategies for implementing cascade screening with 220 individuals with FH (i.e., probands) per arm identified from a large northeastern health system. The primary implementation outcome is reach, or the proportion of probands with at least one first-degree biological relative (parent, sibling, child) in the USA who is screened for FH through the study. Our secondary implementation outcomes include the number of relatives screened and the number of relatives meeting the American Heart Association criteria for FH. Our secondary clinical effectiveness outcome is post-trial proband cholesterol level. We will also use mixed methods to identify implementation strategy mechanisms for implementation strategy effectiveness while centering equity.

**Discussion:**

We will test two patient-facing implementation strategies harnessing insights from behavioral economics that were developed collaboratively with constituents. This trial will improve our understanding of how to implement evidence-based cascade screening for FH, which implementation strategies work, for whom, and why. Learnings from this trial can be used to equitably scale cascade screening programs for FH nationally and inform cascade screening implementation efforts for other genetic disorders.

**Trial registration:**

ClinicalTrials.gov, NCT05750667. Registered 15 February 2023—retrospectively registered, https://clinicaltrials.gov/study/NCT05750667.

**Supplementary Information:**

The online version contains supplementary material available at 10.1186/s13012-024-01355-x.

Contributions to the literature

- This trial will test the comparative effectiveness of two patient-facing implementation strategies that promote family cascade screening for familial hypercholesterolemia: an automated health system-delivered strategy versus a strategy delivered by a care navigator from a nonprofit organization.
- Our implementation strategies were co-designed with key constituents using best practices from behavioral economics and implementation science. If the strategies prove effective, they can inform future efforts to improve the diagnosis of familial hypercholesterolemia and other genetic disorders.
- Our equity-focused analyses will elucidate important insights related to implementation strategy mechanisms, particularly among minoritized populations.

## Background

The field of implementation science has emerged to address the seemingly intractable ‘know-do’ gap in healthcare delivery [1]. Recent thought pieces in the wake of the racial reckoning occurring in the USA following the acute stage of the COVID-19 pandemic and the murder of George Floyd highlight the importance of attending to health equity within the context of implementation [2–4]. Furthermore, there is an increasing emphasis on the importance of bringing implementation science insights to increase health equity in cardiovascular disease [5] and genomic medicine [6]—areas that have, to date, been slower to adopt implementation science approaches. In the present study, we will address a major know-do gap in cardiovascular disease health equity that has relevance to other genetic conditions: increasing implementation of family cascade screening for the autosomal-dominant disorder familial hypercholesterolemia (FH), centering populations with documented inequities (Black or African American people, Asian or Asian American people, and women across racial groups) [7, 8]. This hybrid effectiveness-implementation type III randomized controlled trial (RCT) [9] will (1) test the comparative effectiveness of two patient-facing implementation strategies to increase family cascade screening for FH and (2) use mixed methods [10, 11] to examine equitable implementation and elucidate implementation strategy mechanisms with a focus on health equity.

Our trial design innovates and builds upon the implementation science literature in several ways. First, we examine patient-facing implementation strategies that were co-designed and piloted in collaboration with key partner groups to enhance their likelihood of success [12–14]. The design process included rigorous contextual inquiry informed by the updated Consolidated Framework for Implementation Research (CFIR) [15] and multiple rounds of feedback and refinement. Second, we leveraged behavioral economics insights in the design process. Unlike most prior implementation studies—which typically assume that clinicians and patients make decisions that maximize utility—behavioral economics recognizes that human decision-making is constrained by limits on cognitive capacity and the information available, leading humans to act under ‘bounded rationality’ [16]. Bounded rationality leads individuals to rely on common heuristics and biases such as ease of choice when making decisions [17, 18]. The incorporation of insights from behavioral economics in other areas of healthcare has led to strong results with respect to supporting clinician and patient behavior towards more evidence-based care [19–24]. We leverage these insights in the present study to bolster our implementation strategies and maximize their chance for success. Third, we draw upon the updated CFIR [15] to inform our mixed-methods examination of mechanisms that impact our implementation strategies’ effectiveness and the RE-AIM extension for sustainment and equity [25] to inform our implementation outcomes. Our mixed-methods approach is enriched with the Health Equity Implementation Framework [26], which focuses on health equity determinants such as medical mistrust and discrimination. Drawing upon these leading implementation frameworks roots our approach in the latest thinking in the field.

## The public health problem

FH is a common autosomal dominant genetic disorder characterized by markedly elevated low-density lipoprotein cholesterol (LDL-C) from birth onward, leading to an increased risk for premature atherosclerotic cardiovascular disease (ASCVD). An estimated 1.3 million individuals in the USA have FH, but only about 20% have been diagnosed [27, 28]. Children with FH can develop early ASCVD in the first decade of life and, if left untreated, develop major cardiovascular events, often in middle adulthood [29, 30]. Over their lifetimes, individuals with FH have a 10- to 20-times greater risk of a major adverse cardiac event, including myocardial infarction or stroke, and a 24-times greater risk of myocardial infarction by age 40 [27].

Early identification and treatment of FH can reduce ASCVD risk by approximately 80% [27]. Furthermore, as noted, there are documented inequities in the diagnosis and treatment of FH. Black or African American people, Asian or Asian American people, and women across racial groups are diagnosed later on average than white people and men, respectively, and Black or African American individuals and Asian or Asian American individuals with FH are less likely to achieve recommended cholesterol levels than white individuals [7, 8]. To reduce morbidity and mortality in individuals with FH, we must equitably close this diagnosis gap and alert diagnosed individuals to the existence of lifesaving treatments, allowing them to start treatment earlier.

## The evidence-based practice

Family cascade screening (hereafter, ‘cascade screening’)—or identifying, contacting, and screening relatives of someone who has been diagnosed with FH (i.e., a proband)—is a Centers for Disease Control and Prevention (CDC) Tier 1 genomic application with Grade A evidence-based recommendations [31]. Cascade screening focuses healthcare resources on individuals who are identified as being at elevated risk due to heritability patterns. Ideally, it can help identify individuals before they experience premature cardiovascular events and/or before they would otherwise be exposed to screening as part of routine medical care. This is particularly true if a proband has young relatives; although recommended, lipid screening occurs in only 2–22% of children between the ages of 9–11 [32].

Cascade screening has been successfully implemented in many countries worldwide [33–35]. Its most successful implementation, conducted in the Netherlands [36–38], involved sharing contact information for newly diagnosed individuals with a national foundation, which in turn contacted the proband, requested contact information for relatives, then directly contacted relatives who could be screened for FH in their home by field workers or at a local medical center [36]. That program identified 70% of FH cases nationwide.

## Research-to-practice gap

Despite strong evidence and recommendations to implement cascade screening in the USA, it is not routinely done [39–41]. Further, there is limited literature estimating the exact rates of cascade screening in usual care. One of the only available studies, which involved activities beyond what is typically delivered in routine care in most healthcare settings (e.g., proband completed a questionnaire, a genetic counselor took a family history and offered to discuss screening with relatives directly), found that with this enhanced “usual care” approach, only 8.8% of probands had at least one relative screened [42]. Notably, several drivers of this ‘know-do’ gap have been identified and can inform efforts to promote cascade screening. These include costs and insurance coverage for screening; probands or relatives having concerns related to privacy and discrimination, limited knowledge of FH, or low health literacy; challenging family relationships; clinicians lacking awareness or perceiving FH as low urgency; competing demands on clinicians’ time; and challenges with collaboration among clinicians [43, 44].

Importantly, we know even less about the implementation of cascade screening for minoritized populations. A scoping review of the cascade screening literature found that 74% of studies did not report participants’ race and ethnicity, and those that did include this information did not focus on closing inequities [45]. Given low rates of diagnosis of FH in the USA population, and the existing inequities in FH diagnosis and health outcomes among minoritized groups, efforts to equitably increase the implementation of this evidence-based practice are needed.

## The present study

The present study is a collaboration between researchers from the University of Pennsylvania, Northwestern University, and the Family Heart Foundation (FHF), a national nonprofit research and patient advocacy organization. FHF was founded to address gaps in healthcare for individuals with FH and has collaborated on more than 20 papers in peer-reviewed journals, provides web-based educational resources for clinicians and patients, has trained more than 200 Family Heart Patient Ambassadors, hosts an annual scientific meeting and online peer support groups, and offers navigation services to help those with FH understand their diagnosis and access care. The work described here is funded by a National Heart, Lung, and Blood Institute (NHLBI) R61/R33 award. In the 1-year R61 piloting phase, we conducted contextual inquiry, designed the two patient-facing implementation strategies with insights from behavioral economics (one modeled after the successful approach in the Netherlands), and iteratively piloted and refined the strategies based on end-user feedback [46]. Here, we describe the study protocol for the fully powered trial (i.e., R33 phase of the award).

We center health equity in several ways in this study. First, the implementation strategies were designed with equitable implementation in mind; we strove to maximize accessibility and minimize the risk that they would introduce or perpetuate existing inequities in FH diagnosis. Second, while we do not enrich our overall sample of eligible individuals with people from groups with documented inequities in FH diagnosis (because we will recruit the entire sample of eligible probands from one specialty cardiology program within a health system), the eligible proband sample mirrors the prevalence of Black or African American people and Asian or Asian American people in the USA (prevalence of each group is within 2 percentage points of their respective prevalence in the USA population) and has more women (~ 69%) than men. To ensure we center diverse voices—especially those from groups that have documented disparities in FH diagnosis—we will enrich our qualitative interview sampling with individuals from these groups. Third, our second aim focuses entirely on examining whether our implementation strategies achieve equitable reach across several domains (e.g., race, ethnicity, sex, income) and using mixed methods to investigate mechanisms through which our implementation strategies work, while centering equity.

## Methods/design

This paper adheres to the Standards for Reporting Implementation Studies (StaRI; Additional file 1) [47].

This hybrid effectiveness-implementation type III RCT will test two approaches to increasing family cascade screening for FH: (1) an automated health system-mediated strategy that involves text messages and emails sent from the University of Pennsylvania Health System (“Penn Medicine”); and (2) a foundation-mediated strategy that involves initial outreach from Penn Medicine followed by handoff to a care navigator from FHF, modeled after the successful approach used in the Netherlands. The study will be conducted among 440 eligible probands from Penn Medicine and their biological first-degree relatives living in the USA. The study aims to (1) assess the comparative effectiveness of the two implementation strategies at increasing reach of cascade screening for FH (our primary implementation outcome) and other secondary implementation and clinical effectiveness outcomes; and (2) use mixed methods to examine equitable implementation and identify implementation strategy mechanisms, with a focus on health equity. We will also randomize probands to the usual care (UC) arm to descriptively assess the comparative effectiveness of each active arm versus UC via secondary data analyses. We expect to enroll 360 eligible probands in the UC arm. The trial is intentionally pragmatic in nature to mimic real-world conditions; it scores highly on the PRECIS-2 (Additional file 2) [48].

## Regulatory approvals

This trial was published on ClinicalTrials.gov (NCT05750667) on February 15, 2023. The Penn Institutional Review Board (IRB), which will serve as the single IRB, approved the R33 RCT protocol on January 17, 2023 (Protocol #851061). The Northwestern University IRB has established a reliance agreement with the Penn IRB for this study. The study is overseen by a Data Safety and Monitoring Board and an External Advisory Board, which both meet annually.

## Study aims and approach

### Setting

Penn Medicine serves a racially, ethnically, and socioeconomically diverse population across urban, suburban, and rural areas in and around Philadelphia, via six hospitals and approximately 100 community-based practices. Biological first-degree relatives of participating Penn Medicine patients (or former patients) are expected to live in this geographic area and across the USA and receive their own routine medical care across a wide variety of health systems. The Family Heart Foundation is USA-based and serves individuals both nationally and internationally.

### Participants

**Fig. 1:**
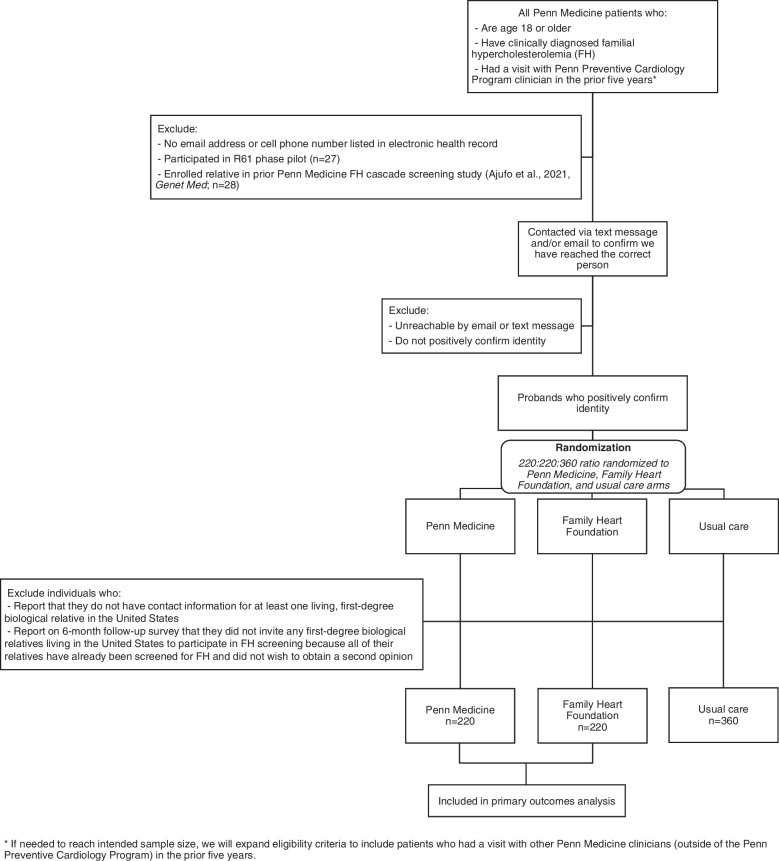
Randomized controlled trial CONSORT diagram

Our RCT will include two types of participants: probands with FH (recruited directly from Penn Medicine) and their first-degree biological relatives living in the USA (referred by probands). In addition, post-RCT qualitative inquiry will include study personnel as well as FHF leadership (recruited directly). We describe study eligibility criteria and rationale in Table 1 and the CONSORT diagram is shown in Fig. 1. Our selection criteria are broad to maximize generalizability. We will draw eligible participants using the methods described below until we accrue 220 eligible probands in each active arm (Penn Medicine, FHF).

**Table 1:** Study eligibility criteria and rationale

Participant group	Criterion type	Criterion	Rationale
Penn Medicine patients diagnosed with familial hypercholesterolemia (FH; “probands”): randomized controlled trial participants	Eligibility criteria for trial enrollment	Age 18 or older	We will exclude children under age 18 from our proband participant group because the health system from which we draw our proband sample (Penn Medicine) does not have pediatric practices and thus, does not typically see children for primary care
		Has clinically diagnosed FH (e.g., have ICD-10 code 78.01 documented in their electronic health record [EHR] or clinician documentation of FH diagnosis in a visit note, both confirmed via chart review)	We will require clinical diagnosis of FH but will not require prior genetic testing for FH for probands to be eligible. Although some probands will have had genetic testing to confirm FH status, we will not require this for several reasons: (a) a diagnosis of FH may be based on clinical assessment alone and does not require a genetic test; (b) up to 50% of individuals with clinically diagnosed FH may not have an identifiable genetic variant; and (c) we wish to avoid exclusion of patients who may have reservations about genetic testing even when it is provided free of charge, including minoritized populations [44, 82, 83]. Furthermore, there are low rates of genetic testing for FH in routine practice; therefore, we have opted to mirror current practice given the pragmatic nature of the study
		Had a visit with a Penn Preventive Cardiology Program clinician in the prior 5 years (whether or not they are engaged in care at the start of the study)	We will limit our inclusion criteria to probands seen by a clinician within the Penn Preventive Cardiology Program—a cohort of clinicians with preventive cardiology expertise who see patients in multiple clinical sites across Penn Medicine—because these individuals are likely to be aware of their FH diagnosis prior to study outreach. In pilot work, we found that many probands with documentation of an FH diagnosis within their electronic health record were not aware of their diagnosis. It would be inappropriate for probands to be alerted to their FH diagnostic status via automated messages delivered by this study. Thus, we will focus our recruitment on individuals seen in the Penn Preventive Cardiology Program, who are likely to be aware of their FH status already. If needed to reach our intended sample size, we will expand our eligibility criteria to include patients who had a visit with other Penn Medicine clinicians (e.g., clinicians from the Penn Medicine Interventional Cardiology Program) We estimate that 5 years would be the timespan for the inclusion of individuals in outreach for quality improvement initiatives conducted by Penn Medicine (i.e., we will mirror what might be done outside of a research context for this study)
		Have an email address and/or cell phone number listed in the EHR	Email address and/or cell phone number are needed for delivery of the automated Penn Medicine-mediated implementation strategy. Based on prior Penn Medicine studies, 90% of patients have cell phones, and nearly all patients who do not have a cell phone listed in their EHR do have an email address listed
		Did not participate in our R61 phase pilot (*n* = 27)	These participants already received an earlier version of the implementation strategies
		Did not enroll a relative in a prior Penn Medicine FH cascade screening study conducted by Ajufo and colleagues (*n* = 28) [42]	These participants already received outreach from Penn Medicine about cascade screening and worked with a genetic counselor to enroll relatives in cascade screening
		Unreachable by email or text message (i.e., delivery failure)	To ensure that health information is shared only with the intended recipient, eligible proband participants will be asked to confirm their identity via text messages and/or email prior to mention of FH. These participants who cannot be reached to confirm identity will not be randomized and thus, will not have a chance to participate
		Do not positively confirm their identity when we first send them a message (i.e., respond ‘no’ or do not respond when we ask if we have reached the correct person)	Similarly, these participants who do not positively confirm identity will not be randomized and thus, will not have a chance to participate
	Exclusion criteria for analyses	Proband reports that they do not have contact information for at least one living, first-degree biological relative in the USA	These probands do not have any relatives that are eligible to complete screening
		Proband reports on a 6-month follow-up survey that they did not invite any first-degree biological relatives living in the USA to participate in FH screening because all of their relatives have already been screened for FH and did not wish to obtain a second opinion	We will exclude probands whose first-degree relatives have all been screened for FH (and do not wish to obtain a second opinion) because these families are not candidates for cascade screening. Because FH screening procedures may vary or evolve over time, we will not exclude relatives who have had previous FH screening but wish to obtain a second opinion
Relatives: randomized controlled trial participants	Eligibility criteria	First-degree biological relative of a participating proband	Although cascade screening ideally includes contacting and screening second-degree relatives, we will focus the finite resources for the present trial on first-degree relatives
		Living in the USA	In this study, we wish to understand cascade screening within the USA context. Additionally, we anticipate that this would be an eligibility criterion for the inclusion of individuals in outreach for quality improvement initiatives conducted by Penn Medicine (i.e., we will mirror what might be done outside of a research context). Finally, the lipid testing options that we will include in this study are only available to those living in the USA
		Age 2 years or older (with contact directed at the caregiver for relatives ages 2–17)	FH guidelines do not recommend lipid screening for children under age two unless both parents are known to have heterozygous FH, so children under two will not be eligible to complete screening through the study
Research team and Family Heart Foundation leadership qualitative interview participants	Eligibility criteria	All staff members involved in deploying the implementation strategies and Family Heart Foundation leadership	n/a

### Evidence-based practice

We operationalize our evidence-based practice of interest (cascade screening) as (1) a first-degree relative completing a lipid panel and (2) clinician review of the lipid panel results and the relative’s medications and personal and family health history to determine whether the relative meets the criteria for FH.

#### Implementation strategies

We describe our implementation strategies and their development, as well as standard practice (i.e., UC) for FH cascade screening at Penn Medicine, in detail in Additional file 3. Briefly, the strategies were developed using a multi-step, iterative process involving key constituent groups (e.g., clinicians, probands, relatives) that included qualitative interviews, cognitive interviews, a focus group, integrating insights from behavioral economics and implementation science into strategy design, and piloting study procedures. We attended to equity and accessibility of our strategies throughout their development.

The content delivered via both strategies will be similar. First, probands will receive educational information about FH and cascade screening from the health system via automated methods. Then, they will be invited to either share similar educational information in the form of a letter with their relatives (‘self-contact’) or share their relatives’ contact information so the study team can contact the relatives directly (‘direct contact’) via automated outreach (Penn Medicine strategy) or phone call from the care navigator (FHF strategy). For both self- and direct contact delivered via both implementation strategies, relatives will receive educational information, be offered options for obtaining a new lipid panel or sharing results from a recent lipid panel, and be invited to complete a telephone consultation call with a study clinician to review lipid panel results. When identified relatives are under age 18, contact will happen with the relative’s caregiver.

Key differences between strategies include delivery modality (automated text messages or emails from the proband’s health system versus telephone calls from a care navigator at FHF) and source of information and assistance (health system vs. a national nonprofit research and patient advocacy organization).

Table 2 describes the implementation strategies using Proctor and colleagues’ reporting recommendations [49].

**Table 2:** Implementation strategy specification

Domain	Strategy: Penn Medicine-mediated	Strategy: Family Heart Foundation-mediated
Actor(s)	Penn Medicine via Way to Health (W2H)	Penn Medicine via Way to Health (used only for initial contact about the study, then participants are given warm hand-off to care navigator from Family Heart Foundation) Family Heart Foundation (FHF) via care navigator
Action(s)	For Penn Medicine patients who have been diagnosed with familial hypercholesterolemia (FH; i.e., the “probands”): Provide education about FH and family cascade screening; explain options^a^ for identifying and contacting living, first-degree biological relatives to invite them to complete screening for FH For relatives: Provide education about FH and cascade screening; explain options for completing screening (i.e., lipid panel); give instructions for completing selected screening option; offer free access to ‘results review’ phone call with an expert clinician	
Target(s) of the action	Probands: Identifying relatives and selecting method(s) to contact relative(s) Relatives: Completing screening and results review phone call	
Temporality	Within 6 months of proband randomization	
Dose	Proband: Approximately 30 blocks of information (containing educational information and/or questions) sent via text message over the course of approximately 1–60 days, depending on proband’s answers and timeliness of responses; or one email linking to a survey containing all of the informational content and questions Relative: *Direct contact*: Approximately 16 blocks of information (containing educational information and/or questions; as described above under ‘Action(s)’ header) sent via text message over the course of approximately 1–30 days, depending on relative’s answers and timeliness of responses; or one email linking to a survey containing all of the informational content and questions *Self-contact:* Proband shares educational ‘Dear Family’ or ‘Dear Caregiver’ letter containing the same content delivered via direct contact (as described above under ‘Action(s)’ header) If a relative chooses to complete screening, they obtain results from a recent lipid panel or a new lipid panel, then complete a results consultation call with the study clinician lasting approximately 20 min	Proband: *W2H initial outreach*: Approximately six blocks of information sent via text message over the course of approximately 1–6 days, depending on proband’s answers and timeliness of responses; or one email linking to a webpage containing the same informational content *FHF care navigator outreach*: One phone call (or more if needed or requested) lasting approximately 15 min total Relative: *Direct contact:* One phone call (or more if needed or requested) lasting approximately 15–30 min total. Discussion covers topics as described above under ‘Action(s)’ header *Self-contact:* Proband shares educational ‘Dear Family’ or ‘Dear Caregiver’ letters containing the same content delivered via direct contact (as described above under ‘Action(s)’ header) If a relative chooses to complete screening, they obtain results from a recent lipid panel or a new lipid panel, then complete a results consultation call with the study clinician lasting approximately 20 min
Implementation outcome(s) affected	Relatives’ completion of screening (i.e., sharing results from recent lipid panel or completing new lipid panel and completing results consultation call with study clinician)	
Justification	Automated messages are a scalable and effective way to encourage health behaviors [50, 51, 84]; participants can engage with information on their own schedule	A foundation-led FH cascade screening program in the Netherlands was highly effective at increasing FH screening [36–38]; personalized contact can address complex barriers

^a^Options for probands to contact relatives through the study are as follows. (1) ‘Direct contact,’ whereby the proband shares relative contact information with Penn Medicine via text message or online survey (Penn Medicine arm) or with the care navigator via phone conversation (Family Heart Foundation arm), and then the relative receives direct outreach via automated messages (Penn Medicine arm) or phone call from the navigator (Family Heart Foundation arm). Probands have the option to give relatives a ‘heads up’ before sharing their contact information with the study. (2) ‘Self-contact,’ whereby the proband receives an informational handout (‘Dear Family’ letter addressed to adult relatives and/or ‘Dear Caregiver’ letter addressed to caregivers of relatives ages 2–17) and tips for discussing cascade screening with relatives

### Recruitment

To ensure that health information is shared only with the intended recipient, eligible proband participants will be asked to confirm their identity via text messages and/or email prior to the mention of FH. Initial proband contact will be delivered via Way to Health (W2H), an evidence-based patient engagement platform [50–52]. Only probands who positively confirm identity will be eligible for randomization to one of the two active study arms (Penn Medicine, FHF) or UC.

### Informed consent

Because proband study activities are considered standard of care and are minimal risk, we have a waiver of informed consent and HIPAA authorization, permitting us to engage with proband participants who are Penn Medicine patients without obtaining informed consent. Adult relative participants for whom the proband selects ‘direct contact’ and who confirm identity will receive a link to (Penn Medicine) or will be read over the phone (FHF) a modified, opt-out consent that describes basic information about the study, why they are being contacted, and instructions for opting-out of participation. Those who opt-out within the designated 72-h window will receive a link to an educational flyer and instructions for what to do if they change their mind and would like to participate in the screening program in the future. All adult relatives who elect to obtain a lipid panel through the study will complete verbal (over the phone) or electronic informed consent prior to testing. For youth relatives (ages 2–17), after the caregiver positively confirms identity, we will share initial program information, including FH education and the offer of free FH screening. If the caregiver expresses a desire to proceed with FH screening for their child, we will obtain electronic or verbal informed consent from the caregiver (or legal guardian if the caregiver is not the legal guardian) and assent from youth ages 7–17. For children ages 2–7, we have obtained a waiver of assent. All qualitative interview participants (probands, relatives, study personnel, FHF leadership) will provide verbal informed consent before beginning the interview.

### Randomization

Immediately after a proband confirms their identity, the W2H platform will randomize them. Enrollment will continue until 220 eligible probands each are enrolled in the two active arms (Penn Medicine, FHF) and 360 eligible probands are enrolled in UC.

### Data collection procedures

#### Active arms baseline data collection approach

Detailed data on automated contact with all study participants will be collected via W2H, and all navigator contacts in the FHF arm will be documented in REDCap [53]. The study clinician in each arm will also use REDCap to collect the data needed to inform study outcomes. The duration of these activities for both probands and relatives will last approximately 1 week to 6 months, depending on participant response speed and activity completion. Probands and relatives will not receive compensation for completing these study activities.

#### Usual care baseline data collection approach

UC probands that positively confirm identity will receive an invitation to complete a brief (~ 1 min) one-time survey inquiring about their preferences for receiving health information. We include the survey to avoid both identity confirmation outreach without follow-up (which would deviate from typical health system practice) and contamination between conditions (e.g., calling attention to FH status). UC probands will be offered the chance to enter a drawing to win one $100 gift card in exchange for completing the survey. Contact data will be captured by W2H and baseline survey data will be captured via REDCap.

#### Six-month follow-up survey

Probands in all three arms will receive text messages and/or emails via W2H 5 months post-randomization, inviting them to complete an online survey. Active arm participants will be asked to report on (a) the number of ‘self-contact’ relatives identified during initial outreach with whom they spoke about FH cascade screening or shared the educational letter; and (b) how many other, not-previously-identified relatives with whom they spoke about FH cascade screening or shared the educational letter. UC probands will be asked how many relatives they communicated with about FH and cascade screening since their date of randomization. All probands will receive $25 for completing this survey. Probands who report communicating with one or more relatives about FH and cascade screening (‘self-contact’ option in the active arms) will be invited to speak with their relatives to ask if they completed FH screening, and if so, to share the results. Probands will be sent a second survey (Part 2 Survey) 7 days after they complete the first survey; it will ask them to report what they learned after speaking with their relatives. Probands will receive an additional $25 for completing this second survey. Surveys will close to data collection 6 months after the proband’s randomization date.

#### Twelve-month lipid panel

Probands in the active arms (Penn Medicine, FHF) will be contacted via W2H 11 months after their randomization date and invited either to (a) complete a free lipid panel through the study or (b) obtain a lipid panel on their own (e.g., through their primary care provider). Participants will receive $25 for sharing results. Data collection for this lipid panel will close at 13 months after the proband’s randomization date. Additionally, if probands do not have a baseline lipid panel in their Penn Medicine electronic health record (EHR; defined as within the 5 years preceding their randomization date), they will be invited to share results from any lipid panel obtained outside of Penn Medicine during that time, if available. We include this clinical outcome because we anticipate that participating in either of the two active implementation approaches may result in better proband awareness and understanding of FH, which could directly lead to initiation or intensification of treatment.

### Study timeline

Enrollment in the RCT will be conducted on a rolling basis until eligible participant targets are reached. Enrollment is anticipated to last approximately 3 years. Each individual proband’s participation will last approximately 12 months, from initial enrollment through completion of a 12-month lipid panel.

## Aim 1: Assess the comparative effectiveness of the two active implementation strategies at increasing reach of cascade screening for FH (primary) and other implementation and clinical effectiveness outcomes (secondary)

We detail our Aim 1 outcomes in Table 3.

**Table 3:** Aim 1 study outcomes

Outcome type	Outcome level	Study arms included in analysis	Outcome name	Outcome description
Implementation outcome	Primary	Penn Medicine, Family Heart Foundation	Reach	The proportion of eligible probands (i.e., Penn Medicine patients diagnosed with familial hypercholesterolemia or “FH”) who have at least one relative who is screened for FH within 6 months of the proband’s randomization date. We define ‘completed screening’ in the Penn Medicine and Family Heart Foundation study arms as relatives sharing lipid panel results and completing a results consultation call with a study clinician. This information is documented by the study clinician during the call in REDCap
	Secondary	Penn Medicine, Family Heart Foundation, Usual care	Comparison of reach to usual care	To compare reach in the active study arms (i.e., Penn Medicine and Family Heart Foundation) to that in the usual care arm, we will use a secondary measure of reach: the proportion of eligible probands who self-report their relatives’ completion of screening for FH, as reported on the Part 2 Survey that follows the 6-Month Follow-Up Survey
	Secondary	Penn Medicine, Family Heart Foundation	Engagement	The proportion of probands who respond to at least one outreach attempt at any point after positive identity confirmation, as identified via Way to Health (W2H) reports and Family Heart Foundation care navigator contact logs
	Secondary	Penn Medicine, Family Heart Foundation	Number of relatives screened	The number of relatives who both: (a) share lipid panel results and (b) complete a telephone consultation with a study clinician. We will use the same methods as those used for our primary outcome of reach to collect both (a) and (b)
		Penn Medicine, Family Heart Foundation	Number of relatives meeting criteria for FH	The number of relatives who meet American Heart Association clinical criteria for FH, defined as adults with untreated low-density lipoprotein cholesterol (LDL-C) of ≥ 190 mg/dL or children with untreated LDL-C of ≥ 160 mg/dL [27], within 6 months of proband randomization, as assessed by the study clinician conducting the results consultation call with the relative
Clinical effectiveness outcome	Secondary	Penn Medicine, Family Heart Foundation	Proband LDL-C	Proband LDL-C on a lipid panel completed between 11 and 13 months after proband randomization
	Exploratory	Penn Medicine, Family Heart Foundation	Change in proband LDL-C	Change in proband LDL-C from baseline to the lipid panel conducted between 11 and 13 months after proband randomization. Baseline LDL-C will be collected from a lipid panel conducted anytime in the 5 years preceding proband randomization. If there are multiple lipid panels available, we will use the most recent. While we will collect lipid panel results from up to 5 years preceding the proband’s randomization date (i.e., 5 years is the upper bound), we will reevaluate this cutoff point for ‘baseline’ LDL-C data once all data have been collected but analysis has not begun, and will define a cutoff point based on what point will give us the most complete data but is still reflective of a true ‘baseline’ at the time of proband randomization (i.e., balancing recency of results with the completeness of the baseline data)

### Primary hypothesis

The FHF-mediated strategy will result in greater reach than the Penn Medicine-mediated strategy. Secondary outcomes focus on hypothesis generation.

## Aim 2: Use mixed methods to elucidate implementation strategy mechanisms, with a focus on health equity and examine equitable implementation

In this aim, we focus on examining equitable implementation of cascade screening. In Aim 2a, we will conduct qualitative interviews to understand mechanisms, with a specific emphasis on elucidating drivers of implementation strategy effectiveness. In Aim 2b, we will quantitatively investigate differential implementation strategy effectiveness and potential effect modifiers (i.e., race, ethnicity, sex, area deprivation index [54] and/or census tract, and insurance status); and descriptively explore differential strategy effectiveness by income, gender, and medical mistrust.

### Participants and procedures

#### Qualitative

We will use purposive sampling [55] to recruit probands and relatives who participated in each active study arm. Probands will be sampled by the primary implementation outcome (success or failure, defined by reach) to understand both why strategies worked and why they did not work. We plan to interview a total of 60 participants (20 probands each per condition for the active arms: 10 successful, 10 unsuccessful; and 10 successful relatives from each active arm). We expect this sample size will be sufficient to reach data saturation but will continue interviews until saturation is achieved [56, 57]. For proband and relative interviews, we will oversample from groups that have been underdiagnosed and undertreated for FH, including Black or African American people, Asian or Asian American people, and women across racial groups, to maximize variation and ensure that diverse experiences are represented [7, 58–61]. We will also enroll 8–12 individuals from the research team and FHF leadership to understand their perspectives on the mechanisms through which the implementation strategies worked and to inquire about the potential for scale-up in each arm.

To understand mechanisms related to the two implementation strategies, we will develop an interview guide informed by the updated CFIR [15] and enriched with constructs from the Health Equity Implementation Framework [26]. The guide will query around specific mechanisms through which our implementation strategies operate, using the updated CFIR to identify key mechanisms at multiple ecological levels. We will also include questions about social and structural factors that may contribute to health inequities such as experiences of discrimination, lack of healthcare access, and language barriers; how these relate to the success of the implementation strategies; and if they differ across populations. During the interview, we will also verbally administer the 12-item Group-Based Medical Mistrust Scale (GBMMS) [62]. The GBMMS asks questions related to ethnic and/or racial-based medical mistrust or suspicion of healthcare systems and healthcare professionals, as well as perceptions of treatment provided to individuals in the participant’s ethnic or racial group. The GBMMS has demonstrated strong validity and reliability in previous studies (α = 0.87–0.88) and has been validated specifically in Black and Latine women and Black men [62–66]. Finally, the interview guide will include questions about the participant’s income and gender identity. Probands who complete the interview will receive $25.

All interviews with probands and relatives will be conducted after cascade screening data collection has ended to avoid any influence of interview participation on our primary study outcomes. The research team and FHF leadership interviews will be completed after all primary study outcomes have been collected. Interviews will be audio-recorded and professionally transcribed, then loaded into NVivo software (QSR International) for data management and analysis.

#### Quantitative

We will gather basic sociodemographic and clinical information via the Penn Medicine EHR (probands only; i.e., race, ethnicity, sex, address, and insurance status) and via self-report (probands and relatives; i.e., income, gender, and medical mistrust) for those who complete a qualitative interview. We will identify the area deprivation index [54] and/or census tract for each proband based on their address.

#### Outcomes

The outcomes of our mixed-methods approach will be identifying mechanisms through which our implementation strategies worked, particularly those that might explain differential strategy effectiveness across minoritized populations, and exploring effectiveness across groups of individuals experiencing inequities in FH.

#### Hypothesis

We hypothesize that we will observe signals that the FHF-mediated strategy will be more effective overall and in populations experiencing inequities, given that it is outside the health system and facilitated by a care navigator.

### Sample size calculation

Our power analyses are based upon the implementation outcome of reach, consistent with best practices for hybrid effectiveness-implementation type III studies [67]. The power calculation is based on the two-sided *Z*-test at a 0.05 significance level and calculated using R (The R Foundation). We have 80% power to detect a difference of 10 percentage points between the active arms (20% for FHF, 10% for Penn Medicine) with 220 probands in each arm. This threshold (10 percentage points) was identified by health system leadership and clinicians as a clinically meaningful difference in a prior large, pragmatic RCT conducted by members of this study team [68], and other trials have also used this threshold to signify clinical significance [69–72]. Given the relevance of findings pertaining to the difference between each active arm and UC to decisionmakers at Penn Medicine and other health systems, we incorporate this comparison as a secondary analysis. With 360 participants in the UC arm, our power for detecting a difference of five percentage points (5% for UC, 10% for Penn Medicine) is 56%. Aim 2 analyses are exploratory; thus, we do not include a power analysis for these research questions.

### Data analysis

#### Aim 1

For testing hypotheses related to our primary implementation outcome of reach (yes/no), we will conduct two-sample *Z*-tests and report the proportion of reach and 95% confidence intervals in each arm. We have a priori specified proband covariates of interest that we will include in our analyses: age; FH genetic test results (if available); whether the proband previously had a ‘medical genetics’ visit with the Penn Preventive Cardiology Program (described in detail in Additional file 3); number of contacts with Penn Medicine in the past 2 years; prior participation in FH studies and quality improvement initiatives at Penn Medicine; and date of FH diagnosis. If study arms are imbalanced on other variables, we will control for those in the analysis. To assess for imbalance in potential confounding variables on the dependent variable, we will conduct a logistic regression analysis with a binary variable for reach (1 = yes, 0 = no) as the outcome and a binary variable indicating implementation strategy (1 = FHF, 0 = Penn Medicine) as the covariate of interest, adjusting for these variables. The odds ratio parameter for the binary study arm indicator approximates the ratio between the proportion of reach in the two active arms, and we will assess whether the odds ratio parameter is significantly greater than one. A study in the UK found that for cascade screening for FH to be cost-effective, at least two relatives should be screened per proband [73]. Thus, if data allow, we will conduct an exploratory sensitivity analysis that repeats the above analysis with reach redefined as ‘whether an eligible proband had *at least two* relatives who completed FH screening.’ For continuous outcomes (number of relatives screened, number of relatives meeting American Heart Association criteria for FH [27], proband LDL-C), we will conduct similar analyses but substitute a two-sample t-test and linear regression models. All tests will be two-sided at the 0.05 significance level. For missing data, we will use multiple imputation [74].

#### Aim 2

##### Qualitative analysis

Analysis will be guided by an integrated approach that includes identification of a priori attributes (i.e., constructs from the updated CFIR, Health Equity Implementation Framework, and behavioral economics) and modified grounded theory, which provides a rigorous, systematic approach to identifying emergent codes and themes [75]. This integrated approach uses an inductive process of iterative coding. After the initial exploration of data, a comprehensive coding scheme will be developed and applied to all data to produce a fine-grained descriptive analysis. A sample of transcripts will be separately coded and their application of the coding scheme compared to assess the scheme’s reliability. Any disagreements in coding will be resolved through team discussion.

##### Quantitative analysis

We will conduct stratified analysis by repeating the Aim 1 analysis separately in subgroups defined by each candidate effect modifier (i.e., race, ethnicity, sex, area deprivation index and/or census tract, and insurance status). This will allow us to examine the difference in effect sizes (e.g., difference in reach) between subgroups. To test the significance of such a difference, for the primary outcome, we will fit a logistic regression model that uses reach status (yes/no) as the outcome variable and includes three covariates: a binary variable indicating the study arm (1 = FHF, 0 = Penn Medicine), the effect modifier (e.g., race), and a cross term between the study arm and effect modifier. A significant modification effect is indicated if the p-value for the cross-term by the Wald test is less than 0.05. We understand that power is limited given the sample size; therefore, we will carefully examine the size and direction of the effect and may use a significance threshold of 0.1. We will also perform interaction analyses for the secondary outcomes, substituting linear regression models for continuous variables (e.g., LDL-C). Lastly, we will conduct exploratory, descriptive analyses to understand the differential effects of participant income, gender, and medical mistrust.

## Discussion

This hybrid effectiveness-implementation type III RCT is a collaborative effort between researchers from the University of Pennsylvania, Northwestern University, and a nonprofit research and advocacy organization, the Family Heart Foundation. The automated Penn Medicine-mediated strategy will be delivered via text messages and/or email and the FHF-mediated strategy will be delivered by a care navigator. In the trial, we will evaluate the comparative effectiveness of the two implementation strategies’ impact on the reach of cascade screening for FH. We will also explore which strategies are sufficient to support change, for whom, and why; and will specifically investigate mechanisms with a focus on health equity.

This study has several strengths. First, health equity has historically been underemphasized in implementation science [2–4] but is a crucial element of this trial given potential barriers to cascade screening, such as medical mistrust, that may be of particular concern to individuals and groups who have had negative experiences with healthcare or healthcare systems and/or experienced structural or systemic racism [76]. We took several steps to advance our goal of closing or not perpetuating existing inequities in FH diagnosis and treatment in this study. We sought feedback from potential end-users when developing our implementation strategies and kept accessibility, acceptability, appropriateness, and feasibility of our implementation strategies at the forefront when designing our materials to maximize the likelihood that they will achieve equitable reach across participant groups. Furthermore, we focus Aim 2 on examining our data for equitable reach in cascade screening outcomes and utilizing mixed methods to identify mechanisms driving differential implementation strategy effectiveness, oversampling from groups with documented inequities in FH diagnosis for our qualitative interviews. Second, our strong partnership with the Family Heart Foundation allows us to test a foundation-mediated implementation strategy that mirrors the highly successful model from the Netherlands [36–38]. Finally, we will explore mechanisms driving our implementation strategies’ effectiveness—something that is not yet standard in implementation science studies [77–79].

This study also has a few limitations. First, although cascade screening ideally includes contacting and screening second-degree relatives [80, 81], we will focus the finite resources for the present trial on first-degree relatives. Future studies may apply the present study’s learnings to cascade screening for second-degree relatives. Second, we will not require genetic testing for FH for probands who are enrolled in the trial since genetic screening is not yet standard practice in the USA, not all individuals with FH have an identifiable mutation, and genetic screening may not be acceptable or of interest to some individuals even when it is provided free of charge [44]. This may make it more difficult to compare our findings to those of other studies that utilize genetic testing in their approach. Third, our implementation strategy materials will only be available in English, although probands will be invited to speak directly with their relatives, guided by the educational letter, in whatever language they prefer. Fourth, while relatives must live in the USA to be eligible to participate, we will share educational information about FH and cascade screening that can be shared with relatives regardless of where they live.

This study will test rigorously developed implementation strategies and will help answer important questions related to which strategies work, for whom, and why. Its results will be poised to guide future wide-scale implementation—both within and outside of large healthcare systems—of cascade screening for FH and other autosomal dominant genetic conditions, such as hypertrophic cardiomyopathies, arrhythmic disorders, Lynch syndrome, and gene mutations implicated in cancer risks such as BRCA1 and BRCA2. This study has aspired to center equity at every stage and will be able to answer important questions related to the equitable implementation of cascade screening. Learnings from this study can be taken to scale nationally by healthcare systems and/or by the Family Heart Foundation to save lives.

### Supplementary Information

**Additional file 1.** Standards for Reporting Implementation Studies checklist. Denotes pages on which each reporting checklist item can be found in the manuscript.

**Additional file 2.** Assessment of degree to which this trial is pragmatic as assessed using the PRECIS-2. Table of each PRECIS-2 domain, the research team’s self-assigned rating, and rationale.

**Additional file 3.** Implementation strategy development and study arm procedures. Detailed description of implementation strategy development and procedures for each study arm.

## Data Availability

Study data have not yet been collected. Once the study is completed, datasets will be available on reasonable request from the corresponding author (RSB).

## Abbreviations

USA: United States of America
FH: Familial hypercholesterolemia
RCT: Randomized controlled trial
CFIR: Consolidated Framework for Implementation Research
LDL-C: Low-density lipoprotein cholesterol
ASCVD: Atherosclerotic cardiovascular disease
CDC: Centers for Disease Control and Prevention
FHF: Family Heart Foundation
NHLBI: National Heart, Lung, and Blood Institute
StaRI: Standards for Reporting Implementation studies
UC: Usual care
IRB: Institutional Review Board
W2H: Way to Health
EHR: Electronic health record
GBMMS: Group-Based Medical Mistrust Scale
