# Supplementary material for: Family cascade screening for equitable identification of familial hypercholesterolemia: study protocol for a hybrid effectiveness-implementation type III randomized controlled trial
Source: Implement Sci. 2024 Apr 9;19:30. doi: 10.1186/s13012-024-01355-x (PMC11003060; doi:10.1186/s13012-024-01355-x)
Supplement: Supplementary file 1 — Additional file 1. Standards for Reporting Implementation Studies checklist. Denotes pages on which each reporting checklist item can be found in the manuscript. [file 13012_2024_1355_MOESM1_ESM.docx]

**Additional File 1. Standards for Reporting Implementation Studies checklist [1].**

| **Checklist item** | | **Implementation strategy** | **Intervention** | **Found on (manuscript page; Section title, if applicable)** |
| --- | --- | --- | --- | --- |
| **Title** | **1** | Identification as an implementation study, and description of the methodology in the title and/or keywords | | 1; Title  6; Keywords |
| **Abstract** | **2** | Identification as an implementation study, including a description of the implementation strategy to be tested, the evidence-based intervention being implemented, and defining the key implementation and health outcomes | | 3; Abstract (Background and Methods) |
| **Introduction** | **3** | Description of the problem, challenge, or deficiency in healthcare or public health that the intervention being implemented aims to address | | 8-9; The public health problem |
|  | **4** | The scientific background and rationale for the implementation strategy (including any underpinning theory, framework, or model, how it is expected to achieve its effects, and any pilot work) | The scientific background and rationale for the intervention being implemented (including evidence about its effectiveness and how it is expected to achieve its effects) | Implementation Strategy:  7-8; Background  10-11; The present study  Intervention:  9-10; The evidence-based practice |
| **Aims and objectives** | **5** | The aims of the study, differentiating between implementation objectives and any intervention objectives | | 12; Methods/Design  14; Evidence-based practice  18-19; Aim 1  19-21; Aim 2 |
| **Methods: description** | **6** | The design and key features of the evaluation (cross referencing to any appropriate methodology reporting standards) and any changes to study protocol, with reasons | | 12; Methods/Design  16-18; Data collection procedures |
|  | **7** | The context in which the intervention was implemented (consider social, economic, policy, healthcare, organisational barriers and facilitators that might influence implementation elsewhere) | | 13; Setting |
|  | **8** | The characteristics of the targeted “site(s)” (locations, personnel, resources, etc) for implementation and any eligibility criteria | The population targeted by the intervention and any eligibility criteria | Implementation Strategy:  11; The present study  13; Setting and Participants  14-15; Implementation strategies and Recruitment  Intervention:  13-14; Participants |
|  | **9** | A description of the implementation strategy | A description of the intervention | Implementation Strategy: 14-15; Implementation strategies and Additional File 3  Intervention: 14; Evidence-based practice |
|  | **10** | Any subgroups recruited for additional research tasks, and/or nested studies are described | | 19-21; Aim 2 |
| **Methods: evaluation** | **11** | Defined pre-specified primary and other outcome(s) of the implementation strategy, and how they were assessed. Document any pre-determined targets | Defined pre-specified primary and other outcome(s) of the intervention (if assessed), and how they were assessed. Document any pre-determined targets | Implementation Strategy and Intervention: Table 3  21; Outcomes  21-22; Sample size calculation |
|  | **12** | Process evaluation objectives and outcomes related to the mechanism(s) through which the strategy is expected to work | | 19-21; Aim 2 |
|  | **13** | Methods for resource use, costs, economic outcomes, and analysis for the implementation strategy | Methods for resource use, costs, economic outcomes, and analysis for the intervention | n/a |
|  | **14** | Rationale for sample sizes (including sample size calculations, budgetary constraints, practical considerations, data saturation, as appropriate) | | 19-20; Participants and procedures: Qualitative  21-22; Sample size calculation |
|  | **15** | Methods of analysis (with reasons for that choice) | | 22-24; Data analysis |
|  | **16** | Any a priori subgroup analyses (such as between different sites in a multicentre study, different clinical or demographic populations) and subgroups recruited to specific nested research tasks | | 19-21; Aim 2  22-24; Data analysis |
| **Results** | **17** | Proportion recruited and characteristics of the recipient population for the implementation strategy | Proportion recruited and characteristics (if appropriate) of the recipient population for the intervention | n/a |
|  | **18** | Primary and other outcome(s) of the implementation strategy | Primary and other outcome(s) of the intervention (if assessed) | n/a |
|  | **19** | Process data related to the implementation strategy mapped to the mechanism by which the strategy is expected to work | | n/a |
|  | **20** | Resource use, costs, economic outcomes, and analysis for the implementation strategy | Resource use, costs, economic outcomes, and analysis for the intervention | n/a |
|  | **21** | Representativeness and outcomes of subgroups including those recruited to specific research tasks | | n/a |
|  | **22** | Fidelity to implementation strategy as planned and adaptation to suit context and preferences | Fidelity to delivering the core components of intervention (where measured) | n/a |
|  | **23** | Contextual changes (if any) which may have affected outcomes | | n/a |
|  | **24** | All important harms or unintended effects in each group | | n/a |
| **Discussion** | **25** | Summary of findings, strengths and limitations, comparisons with other studies, conclusions and implications | | 24-26; Discussion |
|  | **26** | Discussion of policy, practice and/or research implications of the implementation strategy (specifically including scalability) | Discussion of policy, practice and/or research implications of the intervention (specifically including sustainability) | Implementation Strategy and Intervention: 26; Discussion |
| **General** | **27** | Include statement(s) on regulatory approvals (including, as appropriate, ethical approval, confidential use of routine data, governance approval), trial or study registration (availability of protocol), funding, and conflicts of interest | | 11; The present study  13; Regulatory approvals  37-38; Competing interests and Funding |

**Reference.**

1. Pinnock H, Barwick M, Carpenter CR, Eldridge S, Grandes G, Griffiths CJ, et al. Standards for reporting implementation studies (StaRI) statement. BMJ. 2017:i6795. doi:10.1136/bmj.i6795.
