# Supplementary material for: Family cascade screening for equitable identification of familial hypercholesterolemia: study protocol for a hybrid effectiveness-implementation type III randomized controlled trial
Source: Implement Sci. 2024 Apr 9;19:30. doi: 10.1186/s13012-024-01355-x (PMC11003060; doi:10.1186/s13012-024-01355-x)
Supplement: Supplementary file 2 — Additional file 2. Assessment of degree to which this trial is pragmatic as assessed using the PRECIS-2. Table of each PRECIS-2 domain, the research team’s self-assigned rating, and rationale. [file 13012_2024_1355_MOESM2_ESM.docx]

**Additional File 2. Assessment of degree to which this trial is pragmatic as assessed using the PRECIS-2 [1].**

| **Domain** | **Score** | **Rationale** |
| --- | --- | --- |
| Eligibility | 4 | We will include all Penn Medicine patients with familial hypercholesterolemia (FH) who were seen by a clinician within the Penn Preventive Cardiology Program, a cohort of clinicians with preventive cardiology expertise who see patients in multiple clinical sites across Penn Medicine, within the prior five years. Given the stage of this research, we will limit our inclusion criteria to patients seen within this program (across many Penn Medicine clinical sites) because these patients are likely to be aware of their FH diagnosis. If the implementation strategies being tested in this study are found to be effective in this population, future research could examine how to deploy the strategies in the entire Penn Medicine population of patients with FH (i.e., “probands”). If needed to reach our intended sample size, we will expand our eligibility criteria to include patients who had a visit with other Penn Medicine clinicians (outside of the Penn Preventive Cardiology Program) in the prior five years.  Probands seen within the Penn Preventive Cardiology Program who could logistically receive the implementation strategies will be eligible to receive them with the exception of our exclusion criteria of: probands who do not have any living, first-degree biological relatives who reside in the United States; probands who enrolled a relative in a prior Penn Medicine FH cascade screening study conducted by Ajufo and colleagues [2]; and probands who participated in our team’s pilot work for this trial. Similarly, we will include only exclusion criteria for relatives that are logistically needed for delivery of the implementation strategies with the exception of: relatives must live in the United States. |
| Recruitment | 4 | Study recruitment procedures were designed to be compatible with what would be possible within routine healthcare at Penn Medicine (e.g., automated outreach), with the exception of one staff phone call to eligible probands. |
| Setting | 4 | As noted above, eligible probands will be recruited from a specialty program that spans many clinical sites across Penn Medicine. The implementation strategies will be delivered via the same venues as Penn Medicine usual care outreach (i.e., contacting participants by text messages and/or email). |
| Organization | 4 | The study team – specifically, study clinicians and Family Heart Foundation care navigator – have more expertise in FH than most clinicians delivering usual care in non-specialty contexts at Penn Medicine. This is a deliberate component of a centralized outreach strategy. These study team members have approximately equal expertise to those delivering usual care in the Penn Preventive Cardiology Program and other related specialty contexts (e.g., genetic counseling) at Penn Medicine. |
| Flexibility – delivery | 5 | The trial will be very flexible. Relatives can complete screening for FH in many different ways, and probands and relatives can receive the implementation strategies in many ways. The study team will not conduct strict monitoring or compliance evaluation, nor will they intervene on participants to attempt to improve compliance. |
| Flexibility – adherence | n/a | Adherence is not applicable to the study because every study activity is one-time (e.g., lipid panel, results consultation call with study clinician). |
| Follow-up | 4 | We have one (two-part) follow-up survey that will be sent to probands five months after randomization. This is likely similar to other care or quality improvement initiatives conducted at Penn Medicine. However, the survey is likely longer than surveys delivered by Penn Medicine outside of the research context. Additionally, participants will receive compensation for completing each part of our survey, unlike typical quality improvement initiatives conducted at Penn Medicine. |
| Primary outcome | 5 | Our primary outcome, reach, evaluates the proportion of eligible probands who have at least one relative screened for FH. Completing FH screening has obvious benefit to relatives (i.e., it can identify a need for treatment or rule out FH). |
| Primary analysis | 4 | Our analyses will include minimal exclusion criteria that are mainly logistical in nature (e.g., probands don’t have living relatives that can be screened; probands can’t participate because all of their relatives have already been screened). However, we will also exclude participants who don’t positively confirm identity (because we cannot send health information to those who have not positively confirmed identity) and those who do not have any living, first-degree biological relatives living in the United States. |

Note: A score of 5 represents a very pragmatic approach that is essentially identical to that in usual care and a score of 1 represents a very non-pragmatic approach that is very different (e.g., more restrictive or resource-intensive) than that in usual care.

**References.**

1. PRECIS-2. Health Informatics Centre (University of Dundee); 2016. Available from: <https://www.precis-2.org/>. Cited 2023 Nov 15.

2. Ajufo E, DeGoma EM, Raper A, Yu KD, Cuchel M, Rader DJ. A randomized controlled trial of genetic testing and cascade screening in familial hypercholesterolemia. Genet Med. 2021;23(9):1697-704. doi:10.1038/s41436-021-01192-z.
