# Supplementary material for: Family cascade screening for equitable identification of familial hypercholesterolemia: study protocol for a hybrid effectiveness-implementation type III randomized controlled trial
Source: Implement Sci. 2024 Apr 9;19:30. doi: 10.1186/s13012-024-01355-x (PMC11003060; doi:10.1186/s13012-024-01355-x)
Supplement: Supplementary file 3 — Additional file 3. Implementation strategy development and study arm procedures. Detailed description of implementation strategy development and procedures for each study arm. [file 13012_2024_1355_MOESM3_ESM.docx]

**Additional File 3. Implementation strategy development and study arm procedures.**

**Implementation strategy development.**

We developed our implementation strategies using a multi-step process. First, we conducted qualitative interviews with both Penn Medicine clinicians and probands (i.e., patients who had been diagnosed with familial hypercholesterolemia, or “FH”) and other individuals identified as likely to have FH to understand barriers and facilitators to engaging in family cascade screening for FH and to inquire about specific aspects of our strategies (e.g., receptivity to outreach). Next, we met with individuals at Penn Medicine who had leadership roles and/or insights related to the conduct of our randomized controlled trial (RCT) and sustaining our implementation strategies long-term. Then, we identified behavioral economics and implementation science insights to target barriers and facilitators that emerged from qualitative interviews and drafted the implementation strategies. For example, our messaging provides education about the urgency of getting tested for FH to address present bias [1,2], or the tendency of people to over-weight short-term costs over long-term gains, and incorporates ‘implementation intentions’ to increase FH screening follow-through [3,4]. Next, we conducted cognitive interviews with individuals with FH to obtain feedback on content, language, tone, and format of the draft strategies, and then piloted the strategies with Penn Medicine probands and relatives that probands referred to participate. We conducted post-pilot qualitative interviews to gather feedback from pilot participants. Finally, we gathered feedback from a diverse group of community members (including those with no connection to FH) to gain additional perspectives. We made iterative revisions and updates after receiving feedback at each step of this process. Furthermore, throughout this process, we attended to equity and accessibility of our materials (for example, creating multiple delivery modalities including email-only and audio-only; avoiding jargon and reducing reading level of materials; using language that is inclusive of varying family structures [e.g., adoptive children, half-siblings, non-parent caregivers]; making participation free for all participants; and leveraging insights from the literature regarding assuaging medical mistrust in the framing of our educational materials).

Our finalized implementation strategies aim to (a) educate probands about FH and the importance of cascade screening; (b) assist probands with contacting their relatives to encourage them to complete screening; and (c) assist relatives with completing the intervention (screening and results visit). In both implementation strategies (i.e., Penn Medicine-mediated, Family Heart Foundation [FHF]-mediated), relatives will have the option to use a recent (prior two years) lipid panel or a new lipid panel (obtained at a lab near them with costs covered by the study, obtained via a home test kit provided by the study, or obtained through their healthcare provider and personal insurance coverage, depending on participant preference). When identified relatives are under the age of 18, probands will be asked to provide the child’s name and caregiver’s name and contact information. Key differences between strategies include delivery modality (automated text messages or emails from the proband’s health system [Penn Medicine-mediated] versus telephone calls from a care navigator at the Family Heart Foundation [FHF-mediated]) and source of information and assistance (health system [Penn Medicine-mediated] versus a national nonprofit patient advocacy organization [FHF-mediated]).

**Study arm procedures.**

Because this study has been approved as a pragmatic trial, this is standard of care, and we have a waiver of informed consent and HIPAA authorization for proband participants, we will be able to communicate with participants in a style more representative of typical health system practice, without the inclusion of a consent form before communications can begin. Deploying an approach that is consistent with real world practice will enable us to understand the challenges health systems will face when trying to contact probands about cascade screening. To ensure that health information is shared only with the intended recipient, eligible proband participants in all arms will be asked to confirm their identity via text messages and/or email prior to mention of FH; initial contact refers only to “current or prior care at Penn Medicine.”

Participant contact will be delivered via Way to Health (W2H), an evidence-based patient engagement platform [5-7]. Initial identity confirmation will be by text message. Individuals who are not responsive will receive email outreach and, if needed, will receive up to one automated phone call and one phone call from a member of the study team (calling on behalf of Penn Medicine), asking them to confirm their identity by responding to a text message or email that they have received. Only participants who positively confirm identity will be eligible to be randomized to one of the two active implementation strategy arms (Penn Medicine, FHF) or to usual care.

For both active arms, after positively confirming their identity, probands will receive text messages that contain educational information about FH and that highlight the importance and urgency of family cascade screening. They will have the option to request information by email instead, or to read information all at once via a web link. Initial outreach in both conditions comes from Penn Medicine, given that probands will be given the option to opt out of having their information shared with FHF.

***Penn Medicine-mediated strategy.***

Probands will be offered the choice to share contact information for their relatives so Penn Medicine can reach out to their relatives directly (‘direct contact’) or to contact their relatives themselves (‘self-contact’). Messaging will offer guidance on when one option might be preferable over the other, and probands will have the option to choose different options for different relatives. ‘Direct contact’ relatives will receive a text message via W2H and, after positive identity confirmation, will receive educational information about FH and the urgency of cascade screening. Then, relatives will receive a description of screening options. Relatives with lipid panel results will then be invited to schedule a telephone consult with a Penn Medicine study clinician, who will review the results as well as the relative’s medications and personal and family health history. For ‘self-contact’ relatives, the proband will receive a link to a ‘Dear Family’ letter (for adult relatives) or a ‘Dear Caregiver’ letter (if the relative is aged 2-17). The goal of these materials is to facilitate communication of the same information as the ‘direct contact’ option: (a) educational information, (b) instructions for lipid testing, and (c) instructions for scheduling a telephone consult with the Penn Medicine study clinician.

***Family Heart Foundation-mediated strategy.***

To facilitate participation, individuals in this arm will be automatically referred to FHF seven days after receiving information about FHF and being informed that their contact information will be shared securely with FHF unless they choose to opt out of having their contact information shared. Individuals will receive a reminder of the contact information sharing one day before it is scheduled to occur. Those who do not opt out will receive a text message and/or email from the FHF care navigator with basic educational information about FH and cascade screening (mirroring information already delivered via W2H), and a reminder that a care navigator from FHF will be contacting them to discuss in more detail. Probands will be given the option to select a time for this initial phone call. Then, the care navigator will call the proband to provide more details about FH and cascade screening, and to make a plan for conducting cascade screening with the proband’s eligible relatives. This conversation may involve the care navigator sharing instructions and tips or engaging in problem-solving with the proband to help them make a plan to discuss cascade screening directly with their relatives (i.e., ‘self-contact’) and/or discussing a plan for the care navigator to call the proband’s relatives directly (i.e., ‘direct contact’). Materials will parallel those in the Penn Medicine arm; those preferring to contact relatives on their own (‘self-contact’) will have access to the same ‘Dear Family’ or ‘Dear Caregiver’ letters as in the Penn Medicine arm, and relatives for whom the proband requests direct contact will receive FHF navigator outreach consisting of educational information about FH and cascade screening, instructions for obtaining a free lipid panel if they don’t have results from a recent lipid panel already, and instructions for scheduling a free telephone consult with an FHF study clinician. The content can be modified or expanded upon based on the needs of probands and relatives (e.g., answering specific questions, assuaging concerns about screening, problem-solving logistical barriers), and the care navigator conversations with probands and relatives can span multiple phone calls as needed (e.g., the proband would like to think about which relatives to contact and how before deciding).

***Usual care.***

There is no standard approach for cascade screening with individuals who are diagnosed with FH at Penn Medicine. Within the Penn Preventive Cardiology Program, a cohort of clinicians with preventive cardiology expertise who see patients in multiple clinical sites across Penn Medicine, when a patient presents with the FH clinical phenotype, providers will refer the patient to a genetic counselor within the program, who then assists the patient with obtaining genetic testing. Patients who screen genetically and/or clinically positive for FH are invited to complete a ‘medical genetics’ visit with the genetic counselor, which involves sharing a letter describing recommended next steps for the patient’s (i.e., proband’s) treatment of their FH, taking a detailed family history, and encouraging the patient to inform their biological relatives (identified during the family history discussion) of their FH diagnosis and recommending they complete cascade screening. Thus, the usual care arm in this study will not receive proactive FH-related contact. We describe the contact that probands in the usual care arm will receive for data collection purposes in the main text (‘Data collection procedures’ section).

**REFERENCES.**

1. Camerer CF, Loewenstein G. Behavioral economics: past, present, future. In: Camerer CF, Loewenstein G, Rabin M, editors. Advances in behavioral economics. Princeton University Press; 2004. p. 3-51.

2. Wang Y, Sloan FA. Present bias and health. J Risk Uncertain. 2018;57(2):177-98. doi:10.1007/s11166-018-9289-z.

3. Gollwitzer PM. Implementation intentions: strong effects of simple plans. Am Psychol. 1999;54(7):493. doi:10.1037/0003-066x.54.7.493.

4. Gollwitzer PM, Sheeran P. Implementation intentions and goal achievement: a meta‐analysis of effects and processes. Adv Exp Soc Psychol. 2006;38:69-119. doi:10.1016/S0065-2601(06)38002-1.

5. Asch DA, Volpp KG. On the way to health. LDI Issue Brief. 2012;17(9):1-4. Available from: <https://ldi.upenn.edu/wp-content/uploads/archive/pdf/IssueBrief17_9.pdf>. Cited 2024 Jan 11.

6. Asch DA, Muller RW, Volpp KG. Automated hovering in health care—watching over the 5000 hours. N Engl J Med. 2012;367:1-3. doi:10.1056/NEJMp1203869.

7. Way to Health. University of Pennsylvania. Available from: <https://waytohealth.upenn.edu>. Cited 2023 Nov 15.
